# Effect of Glycyrrhizin on Pseudomonal Skin Infections in Human-Mouse Chimeras

**DOI:** 10.1371/journal.pone.0083747

**Published:** 2014-01-30

**Authors:** Shohei Yoshida, Jong O. Lee, Kiwamu Nakamura, Sumihiro Suzuki, David N. Hendon, Makiko Kobayashi, Fujio Suzuki

**Affiliations:** 1 Department of Internal Medicine, The University of Texas Medical Branch, Galveston, Texas, United States of America; 2 Shriners Hospital for Children, Galveston, Texas, United States of America; 3 Department of Biostatistics, University of North Texas Health Science Center, Fort Worth, Texas, United States of America; University of Cincinnati, United States of America

## Abstract

In our previous studies, peripheral blood lineage^−^CD34^+^CD31^+^ cells (CD31^+^ IMC) appearing in severely burned patients have been characterized as inhibitor cells for the production of β-defensins (HBDs) by human epidermal keratinocytes (NHEK). In this study, the effect of glycyrrhizin on pseudomonal skin infections was studied in a chimera model of thermal injury. Two different chimera models were utilized. Patient chimeras were created in murine antimicrobial peptide-depleted NOD-SCID IL-2rγ^null^ mice that were grafted with unburned skin tissues of severely burned patients and inoculated with the same patient peripheral blood CD31^+^ IMC. Patient chimera substitutes were created in the same mice that were grafted with NHEK and inoculated with experimentally induced CD31^+^ IMC. In the results, both groups of chimeras treated with glycyrrhizin resisted a 20 LD_50_ dose of *P. aeruginosa* skin infection, while all chimeras in both groups treated with saline died within 3 days of the infection. Human antimicrobial peptides were detected from the grafted site tissues of both groups of chimeras treated with glycyrrhizin, while the peptides were not detected in the same area tissues of controls. HBD-1 was produced by keratinocytes in transwell-cultures performed with CD31^+^ IMC and glycyrrhizin. Also, inhibitors (IL-10 and CCL2) of HBD-1 production by keratinocytes were not detected in cultures of patient CD31^+^ IMC treated with glycyrrhizin. These results indicate that sepsis stemming from pseudomonal grafted site infections in a chimera model of burn injury is controllable by glycyrrhizin. Impaired antimicrobial peptide production at the infection site of severely burned patients may be restored after treatment with glycyrrhizin.

## Introduction

*Pseudomonas aeruginosa* burn wound infection frequently develops into sepsis in severely burned patients [1]–[3]. As an initial effector of host antibacterial innate immunities, antimicrobial peptides distributed in skin tissues contribute to control surface (wound) infections [4]–[7]. In severely burned patients, however, sufficient amounts of antimicrobial peptides are not produced in tissues surrounding the burn sites [8]–[10]. The lack of antimicrobial peptide production leads to the invasion of pathogens from surface infections to systemic infections [11]. In our accompanying paper [10], lineage^−^CD34^+^CD31^+^ cells (designated as CD31^+^ IMC) isolated from peripheral blood of severely burned patients (patient CD31^+^ IMC) were shown to be inhibitory on the HBD production by normal human epidermal keratinocytes (NHEK). Lineage^−^CD34^+^ cells isolated from healthy donor peripheral blood were shown to be non-inhibitory on the peptide production by NHEK, and these cells were characterized as lineage^−^CD34^+^CD31^−^ cells. Lineage^−^CD34^+^ CD31^+^ cells were not isolated from healthy donor peripheral blood. CCL2 and IL-10 released from patient CD31^+^ IMC were identified as inhibitory factors for the HBD production by NHEK.

Previously, we have reported that glycyrrhizin protects severely burned mice from lethal doses of *P. aeruginosa* burn wound infections [12]. To test the beneficence of glycyrrhizin in a more clinical setting, in this study, the anti-pseudomonal effect of glycyrrhizin was investigated in a human model of the severe burn injury combined with pseudomonal skin infections. In addition, the effect of glycyrrhizin on the suppressor cell activities of patient CD31^+^ IMC on the HBD-1 production was investigated at the grafted site tissues in the chimeras. A human model of severe burn injury was γ-irradiated NOD-SCID IL-2rγ^null^ mice that were grafted with unburned skin tissues of severely burned patients and inoculated with their syngeneic peripheral blood CD31^+^ IMC (patient chimeras), or grafted with cultured NHEK and inoculated with experimentally induced CD31^+^ IMC (patient chimera substitutes). Experimentally induced CD31^+^ IMC were isolated from peripheral blood of healthy volunteers 24 hours after intense exercise on a treadmill for 1 continuous hour, as previously described [13]. These cells have been shown to transiently appear after intense exercise (heart rate baseline: 130–145 beats per minute) for one continuous hour (first appeared 12 hours after exercising, peaked 24 hours after exercising, and then disappeared).

Glycyrrhizin, a component of licorice root, has been clinically utilized in patients with peptic ulcers, chronic hepatitis, and cirrhosis. Glycyrrhizin has been reported to stimulate various host defense immunities, including inhibition of inflammatory responses [14], augmentation of natural killer cell activities [15], and induction of various type 1 soluble factors (IL-12, IFN-γ CCL3 and CCL5) from immunocompetent cells [16]–[18]. Through immunomodulating activities, glycyrrhizin inhibits the growth of hepatitis virus [19], human cytomegalovirus [20], herpes simplex virus [21], influenza virus [22], [23], HIV [24], [25], coronavirus [26], and *Candida albicans* [27]. For over 40 years, glycyrrhizin has been used clinically to treat infectious hepatitis in Japan.

In the results presented herein, antimicrobial peptides were not produced significantly at grafted site skin tissues in patient chimeras, while the peptides were produced in the same chimeras treated with glycyrrhizin. The compound did not directly stimulate the production and mRNA expression of HBD-1 by epidermal keratinocytes. Sepsis stemming from *P. aeruginosa* skin infection was developed in patient chimeras and patient chimera substitutes treated with saline, while both groups of chimeras treated with glycyrrhizin resisted the infection. Glycyrrhizin may be beneficial against pseudomonal burn wound infection and subsequent sepsis in severely burned patients.

## Materials and Methods

### Ethics Statement

All animal experiments were performed in accordance with protocols approved by The Institutional Animal Care and Use Committee of The University of Texas Medical Branch at Galveston, TX (IACUC approval number: 0404019A). The study was approved by the Institutional Review Board of the University of Texas Medical Branch (IRB approved number: 02-018). Written informed consent for blood sampling was obtained from all adult subjects. For blood sampling from children, written parental consent was obtained. Ethical approval was obtained from the Ethical and Scientific Committee of the University of Texas Medical Branch.

### Animals

Nine- to 12-week-old male NOD-SCID IL-2rγ^−/−^ mice (NOD.Cg-*Prkdc* ^scid^ *Il2rg* ^tm1Wjl^/SzJ mice) purchased from The Jackson Laboratory (Bar Harbor, ME, USA) were used in this study. According to the recent information obtained from the Jackson Laboratory (http://jaxmice.jax.org/events/2011/032411UNIVIL nsg.pdf), we utilized these mice as mice that are deficient in both innate and adoptive immunities (without functional T cells, B cells, and NK cells). Also, these mice are carriers of defective functions (phagocytosis, digestion, antigen presentation, and activation) of macrophages [28]–[31]. Before being used in our experiments, these mice were exposed to whole body γ-irradiation (4 Gy) to deplete neutrophils. Bone marrow cells or peripheral blood cells taken from these mice have been tested morphologically for residual neutrophils after Wright-Giemsa and alkaline phosphatase staining. In the results, neutrophils were not recovered from these mice 1 to 7 days after γ-irradiation, even when they were stimulated with pathogens. After γ-irradiation, these mice were housed in the BSL-2 Animal Facility, and fed autoclaved food and water to avoid infectious complications.

### Thermally Injured Patients

Eighteen burn patients (11 male, 7 female) admitted to the Shriners Hospitals for Children at Galveston and the University of Texas Medical Branch (UTMB) were enrolled in this study. All patients had more than a 30% total body surface area (TBSA) burn (average 56.3±18.2%). The youngest and oldest ages were 2 and 41 years old (average 11.2±9.3), respectively. All patients were subjected to a standard treatment regimen [32]. This regimen consisted of fluid resuscitation, topical antimicrobial agents, and aggressive nutrition support based on estimated energy and protein needed.

#### Reagents, media, and cells

HBD-1 kits were purchased from PeproTech (Rocky Hill, NJ, USA). HBD-1, MBD-1, anti-HBD-1, and anti-MBD-1 antibodies were purchased from Alpha Diagnostic International (San Antonio, TX, USA). Lineage cell depletion kits were purchased from Miltenyi Biotec (Auburn, CA, USA). FITC-conjugated anti-CD34 mAb and PE-conjugated anti-CD31 mAb were purchased from BD Biosciences (Franklin Lakes, NJ, USA) and BioLegend (San Diego, CA, USA), respectively. Recombinant (r) CCL2 and rIL-10 were obtained from PeproTech, and mAbs directed against CCL2 and IL-10 were purchased from BioLegend. Adult normal human epidermal keratinocytes (NHEK) were obtained from Lonza (Walkersville, MD, USA) and propagated in a serum-free keratinocyte growth medium (KMG-2, Lonza) at 37°C. NHEK that underwent a second passage with KMG-2 were stored in liquid nitrogen. NHEK grown from the stored cells (the third passage) were used in this study. RPMI-1640 medium supplemented with 10% FBS, 2 mM L-glutamine, and antibiotics (100 U/ml penicillin and 100 µg/ml streptomycin) was used as culture media for lineage^−^CD34^+^ cells. A Protease Inhibitor Cocktail was purchased from Sigma-Aldrich (St. Louis, MO, USA).

#### Glycyrrhizin

Glycyrrhizin (20β-carboxy-11-oxo-30-norolean-12-en-3β-yl-2-*O*-β-_D_-glucopyranourasyl-α-_D_-glucopyranoic acid) is a triterpenoid saponin in Glychyrrhiza glabra (licorice). A >95% pure glycyrrhizin acid ammonium salt (glycyrrhizin) prepared from the extract of licorice root was supplied from Minophagen Pharmaceutical Co., Ltd, Tokyo, Japan. Glycyrrhizin is converted into glychyrrhetic acid by glycaronidase when it is injected to animals and patients. Glycyrrhetic acid is the active form of glycyrrhizin for various immunomodulating and anti-inflammatory actions. When glycyrrhizin is utilized in experiments, 1,000 µg of the compound was first dissolved in 1 ml of saline or serum free RPMI medium at room temperature (1,000 µg/ml). Then, it was further diluted to the appropriate concentrations with saline or serum free RPMI medium and stored at 4°C until utilized. Based on our previous data [12], [13], 1–100 µg/ml concentrations of glycyrrhizin were added to the tissue cultures, and 1 to 10 mg/kg doses of the compound were administered to the chimeras. These concentrations of glycyrrhizin have been confirmed as non-toxic in many papers [19]–[22].

#### Preparation of CD31^+^ IMC

Lineage^−^CD34^+^CD31^+^ cells (patient CD31^+^ IMC) were prepared from peripheral mononuclear cells of severely burned patients, as previously described [10]. Because large numbers of CD31^+^ IMC were not obtained from severely burned patients, the same cells were experimentally generated from healthy donor peripheral blood cells and utilized in some chimera experiments (to set up experimental conditions and to determine survival effect of glycyrrhizin after *P. aeruginosa* infections). Thus, experimentally induced CD31^+^ IMC were prepared from peripheral blood of healthy volunteers (heart rate baseline: 60–75 beats per minute) subjected to intense exercise on a treadmill (Cybex International, Medway, MA, USA), where their heart-rate was consistently kept elevated to 130–145 beats per minute (metabolic equivalents: 6.5 to 7.8) for one continuous hour. As a result of this exercise program, lineage^−^CD34^+^CD31^+^ cells first appeared 6 or 12 hours after exercising, peaked 24 hours after exercising, and then disappeared [13]. Therefore, lineage^−^CD34^+^CD31^+^ cells isolated from peripheral blood of healthy volunteers 24 hours after intense exercise for 1 hour were utilized as experimentally induced CD31^+^ IMC. Experimentally induced CD31^+^ IMC and patient CD31^+^ IMC were shown to have identical properties for producing IL-10 and CCL2 and inhibiting HBD-1 production by NHEK.

#### Preparation of chimeras

γ-Irradiated NOD-SCID IL-2rγ^−/−^ mice were grafted with unburned skin tissue removed from burn patients (discarded skin from the auto-graft surgery). The function of these discarded skin tissues to produce HBD-1 has been demonstrated [8]. To prepare patient chimeras, under anesthetic, a layer of dorsal skin tissues of γ-irradiated NOD-SCID IL-2 rγ^−/−^ mice was cut (2×2 cm) and folded back to expose the subcutaneous layer. Unburned skin (0.8×0.8 cm) was implanted in a single layer, and the exposed area was surgically closed with absorbable sutures (4-0 Dexon S). To prepare the patient chimera substitute, γ-irradiated NOD-SCID IL-2rγ^−/−^ mice were intradermally inoculated with NHEK (1×10^6^ cells/mouse). Six hours later, these mice were intradermally inoculated with 1×10^6^ cells/mouse of CD31^+^ IMC surrounding the grafting site. HBD-1 was detected in the tissues surrounding the inoculation site of both groups of chimeras without CD31^+^ IMC inoculation until 5 days (NHEK) to 7 days (skin tissue) after grafting.

#### Assays of IMC activities

(1) *In vitro* assay. Two series of *in vitro* assays were performed for the determination of anti-IMC activities of glycyrrhizin. (a) Transwell-cultures: Unburned skin tissues (0.2×0.2 cm) were cultured with patient CD31^+^ IMC (upper chamber) in dual-chamber transwells supplemented with 100 µg/ml of glycyrrhizin. Forty-eight hours after cultivation, culture fluids were harvested and assayed for HBD-1 by ELISA [10]. Also, cells in the lower chamber were analyzed for HBD-1 mRNA by RT-PCR, as previously described [13]. (b) Conditioned medium: Patient CD31^+^ IMC (1×10^6^ cells/ml) that were pre-treated with glycyrrhizin (100 µg/ml) for 12 hours were cultured for 36 hours. Conditioned media were harvested and assayed for their activities to inhibit HBD-1 production by unburned skin. Conditioned media obtained were added to unburned skin (0.2×0.2 cm) cultures (5–20% v/v, 1×10^5^ cells/ml) for 48 hours, and culture fluids harvested were assayed for HBD-1 by ELISA.

(2) *In vivo* assay. The effect of glycyrrhizin on HBD-1 production in patient chimeras or patient chimera substitutes was tested. Glycyrrhizin was administered i.p. to the chimeras 2 and 24 hours after grafting of the peptide-producer cells. Five punch biopsies were randomly excised per chimera from grafted site skin tissues using a sterile biopsy punch (8 mm diameter, Sklar Instruments, West Chester, PA, USA). The depth of the skin biopsy extended all the way to the skeletal muscles of the back (epidermal, dermal, and panniculus carnosus components). Just after excision, the tissues were weighed and rinsed in cold, sterile PBS containing 1% antibiotic mixture (penicillin 100 U/ml, streptomycin sulicoricehate 100 µg/ml) for 2 min. These tissues were then homogenized in 1 ml of cold PBS supplemented with 1% Proteinase Inhibitor Cocktail. The homogenates were centrifuged (1000 *g*, 20 min), and supernatants of the homogenates were assayed for HBD-1 by ELISA. In some confirmation experiments, the same homogenates were assayed for MBD-1 by ELISA.

#### Grafted site infection of P. aeruginosa in chimeras

*P. aeruginosa* strain 180 (American Type Culture Collections, Manassas, VA, USA) was used in this study. *P. aeruginosa* was grown in a brain-heart infusion broth for 18 hours at 37°C. One and 2 days before skin infection, both groups of chimeras were treated s.c. with 100 units of an IgG preparation against anti-murine skin antimicrobial peptide (AMP). Murine AMPs (MDB-1, MBD-2 and MBD-3) were not detected in skin homogenates of either group of chimeras treated with the IgG preparation. Anti-murine skin AMP rabbit IgG was purified from sera of rabbits that were immunized with the murine skin homogenates with Freund’s complete adjuvant, as described previously [33], [34]. An antibody titer of the IgG was measured by its neutralizing activity against recombinant murine β-defensin 1 (rMBD-1). One unit of the IgG titer inactivated 1 µg of MBD-1. The IgG was shown to be active for the neutralization of MBD-2 and MBD-3 activities. The activities of human β-defensins (HBD-1, HBD-2 and HBD-3) were not neutralized by this IgG. After the IgG treatment, γ-irradiated NOD-SCID IL-2rγ^−/−^ mice were shown to be susceptible (100% died) to 10 CFU/mouse of *P. aeruginosa* intradermal (i.d.) infection, while 80% of the same mice treated with saline survived after infection with 10^4^ CFU/mouse of *P. aeruginosa* (**Fig. S1**). In fact, 5 CFU/mouse of *P. aeruginosa* infection was shown to be 1 LD_50_ in γ-irradiated NOD-SCID IL-2rγ^−/−^ mice treated with the IgG, and 10^5^ CFU/mouse of *P. aeruginosa* i.d. infection was shown to be 1 LD_50_ in these mice not treated with the IgG (**Fig. S1**).

To determine the protective effect of glycyrrhizin against *P. aeruginosa* i.d. infection, patient chimeras or patient chimera substitutes that were previously treated with anti-murine skin AMP rabbit IgG (see above) were utilized throughout the infection experiments. Two days after IMC inoculation, 100 CFU/0.1 ml of *P. aeruginosa* (corresponds to 20 LD_50_ severity) was injected i.d. to the grafted skin site of chimeras. The effect of glycyrrhizin was evaluated by the decrease in (i) the mortality rates of patient chimera substitutes and (ii) the growth of pathogen in kidneys of patient chimeras. Kidneys from infected patient chimeras were weighed, and disrupted with an Omni tissue homogenizer with 2 ml PBS. The number of bacteria in the homogenates was measured by a standard colony-counting method and expressed as per gram organ. To determine the percentage of survival, infected mice were monitored twice a day for signs of disease, which typically included piloerection, hunched gait, lethargy and eye discharge. The survival of infected mice was recorded for up to 7 days after infection. The infected mice displaying severe signs of distress (a Quantitative Assessment for Pain & Distress Chart score of 9 or more; decrease in body temperature <35°C, body weight >20% loss, labored breathing, non-responsiveness to cage tapping, failure of grooming and severe eye discharge) were humanely euthanized with ketamine (300 mg/kg, i.p.) and xylazine (30 mg/kg, i.p.), and death was assured by cervical dislocation. The death was recorded as infection induced mortality.

#### Statistical analyses

Data shown in each figure are derived from three independent experiments using different donors. Data are presented as mean ± SEM. Survival curves were analyzed using the Kaplan-Meier method. Chimera groups were statistically compared using a Student’s *t* test. If the *P*-value was less than 0.05, the result was considered to be significant.

## Results

### Susceptibility of Patient Chimera Substitute to P. aeruginosa i.d. Infection and HBD-1 Production at Grafted Site Tissues of these Chimeras

**Figure 1:**
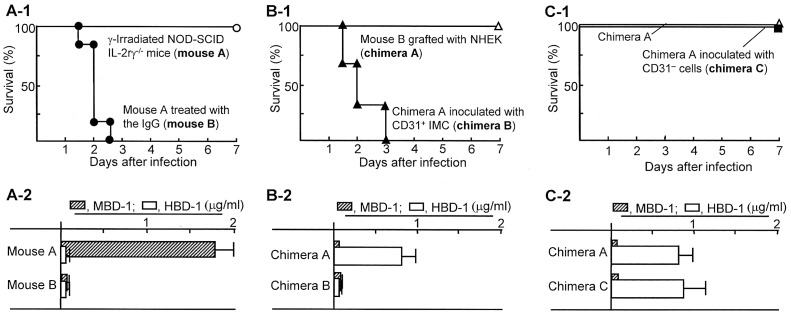
Importance of skin antimicrobial peptides on the resistance of various chimeras to *P. aeruginosa* i.d. infections. **A-1.** γ-Irradiated NOD-SCID IL-2rγ^−/−^ mice treated with (mouse B) or without anti-murine AMP rabbit IgG (mouse A) were i.d. infected with 20 LD_50_ of *P. aeruginosa*. **B-1.** Chimera A (mouse B grafted with NHEK) and chimera B (chimera A inoculated with experimentally induced CD31^+^ IMC, patient chimera substitute) were i.d. infected with 20 LD_50_ of *P. aeruginosa* at the skin surrounding the grafted site tissues. **C-1.** Chimera A (mouse B grafted with NHEK) and chimera C (chimera A inoculated with lineage^–^CD31^−^ cells) were i.d. infected with *P. aeruginosa* in the same fashion. Then, their survivals were observed for a week after the infection. In addition, the antimicrobial peptide production in the grafted site tissues of all groups of chimeras was tested. One day after the IgG treatment (**A-2**) or 2 days after grafting (**B-2, B-2**), 5 biopsies of skin tissues were obtained from the grafting sites. Tissues obtained were homogenized together, and the amounts of HBD-1 and MBD-1 in the homogenates were measured by ELISA. Each infection experiment was performed by a group of 2–3 mice, and it was repeated 3 times. The results obtained were combined and displayed in the figure.

The importance of β-defensins on host resistance against *P. aeruginosa* i.d. infection has been reported [4]–[7]. In our accompanying paper [10], lineage^−^CD34^+^CD31^+^ cells (CD31^+^ IMC) that appeared in peripheral blood of severely burned patients have been shown to be responsible for inhibiting HBD-1 production by NHEK. In the first series of experiments in this study, the role of skin antimicrobial peptides on the resistance of mice against *P. aeruginosa* i.d. infection and subsequent sepsis was confirmed in γ-irradiated NOD-SCID IL-2rγ^−/−^ mice depleted of murine skin antimicrobial peptides. γ-Irradiated NOD-SCID IL-2rγ^−/−^ mice lack functional immunocompetent cells, including T cells, B cells, NK cells, neutrophils, and macrophages. As a result, all these mice depleted of murine skin antimicrobial peptides (mouse B) died after infection with 100 CFU/mouse of *P. aeruginosa*, while all of the mice with the peptides (mouse A) survived even though they were exposed to the same infection (**Fig. 1a-1**). Also, murine antimicrobial peptide (MBD-1) was detected in skin homogenates of γ-irradiated NOD-SCID IL-2rγ^−/−^ mice, while this peptide was not detected in those of the same mice treated with anti-murine AMP rabbit IgG. Human antimicrobial peptide (HBD-1) was not detected in the skin homogenates of γ-irradiated NOD-SCID IL-2rγ^−/−^ mice treated with or without the IgG (**Fig. 1a** **-** **2**). In the next experiments, these mice depleted of the murine peptides were grafted with NHEK (1×10^6^ cells/mouse, i.d.) and inoculated with experimentally induced CD31^+^ IMC at the grafting site tissues. Then, these mice were infected with 100 CFU/mouse of *P. aeruginosa* at the grafted site tissues, and their mortality rates were observed. As a result, all of the chimeras created with NHEK alone (chimera A) survived after *P. aeruginosa* i.d. infection. However, all of the chimeras created with NHEK grafting and experimentally induced CD31^+^ IMC inoculation (chimera B) died within 3 days of the same infection (**Fig. 1b-1**). All chimera B without any infections with *P. aeruginosa* survived for 2 weeks or more. HBD-1 was detected in homogenates of grafted site tissues of chimera A, while HBD-1 was not detected in grafted site skin homogenates of chimera B (**Fig. 1b** **-** **2**). These results indicate that host antibacterial resistance of chimeras created with NHEK against *P. aeruginosa* i.d. infection is completely inhibited by experimentally induced CD31^+^ IMC. Chimera C, created with healthy donor lineage^−^CD34^+^CD31^−^ cells, were shown to be resistant against 100 CFU/mouse of *P. aeruginosa* i.d. infection (**Fig. 1c-1**). As shown in chimera C, the amount of HBD-1 produced in chimera A was not influenced by healthy donor peripheral blood lineage^−^CD34^+^CD31^−^ cells (**Fig. 1c** **-** **2**), indicating that lineage^−^CD34^+^CD31^−^ cells do not suppress HBD-1 production. The results shown in Figs. 1a, 1b, and 1c indicate that skin antimicrobial peptides (a, murine peptides; b and c, human peptides) are very important in the resistance of mice to *P. aeruginosa* i.d. infections, and CD31^+^ IMC are responsible for inhibiting HBD-1 production by skin tissues.

**Figure 2:**
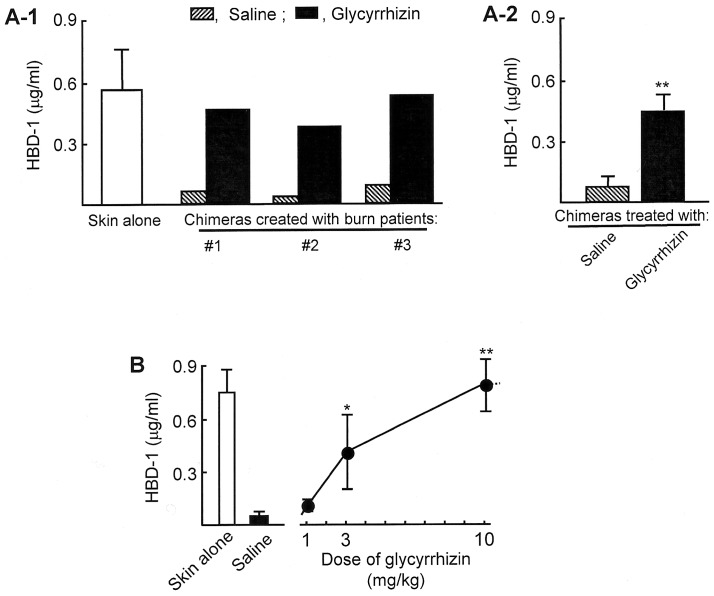
HBD-1 production in patient chimeras treated with glycyrrhizin. **A.** Patient chimeras created with burn patient #1∼#3 unburned skin tissues and their CD31^+^ IMC were treated i.p. with glycyrrhizin (10 mg/kg) 6 and 24 hours after the IMC inoculation. As a control, the chimeras were treated with saline (0.2 ml/mouse). Twenty-four hours after the final glycyrrhizin treatment, 5 skin biopsies were obtained from grafted site tissues, homogenized together, and assayed for HBD-1 by ELISA. A white bar shows the results obtained from a group of mice grafted with skin alone. Fig. 2A-1 shows 3 independent experiments performed using skin and blood specimens from 3 patients, and Fig. 2A-2 shows mean ± SEM of the results shown in Fig. 2A-1. **P<0.01 vs saline-treated control. **B.** The recovery of HBD-1 production in patient chimeras treated with glycyrrhizin. The chimeras created with unburned skin tissues from patients #4∼#7 and their CD31^+^ IMC were treated twice with 1 to 10 mg/kg of glycyrrhizin. As controls, the chimeras were treated with saline (0.2 ml/mouse). Twenty-four hours after the treatment, 5 skin biopsies were obtained from grafted site tissues, homogenized together, and assayed for HBD-1 by ELISA. A white bar shows the results obtained from a group of mice grafted with skin alone (mean ± SEM of the 4 independent experiments). *P<0.05; **P<0.01 vs saline-treated control.

### Effect of Glycyrrhizin on HBD-1 Production in Patient Chimeras

Patient chimeras created with unburned skin tissues from patients #1∼#3 and their respective CD31^+^ IMC were treated twice (6 and 24 hours after the IMC inoculation, i.p.) with glycyrrhizin. One day after the final treatment, 5 skin biopsies obtained from the chimera’s skin grafted sites were homogenized together in 1 ml PBS and assayed for HBD-1 by ELISA. As a result, HBD-1 was not detected in skin samples of any patient chimeras. However, peptide was detected in the chimeras treated with 10 mg/kg of glycyrrhizin at levels produced in γ-irradiated NOD-SCID IL-2rγ^−/−^ mice grafted with patient unburned tissues (**Figs. 2a and 2b**). In patient chimeras treated with 3 mg/kg of glycyrrhizin, 0.45 µg/ml of HBD-1 was detected in their skin homogenates. The impaired peptide production in patient chimeras was not influenced by 1 mg/kg dose of glycyrrhizin treatment (**Fig. 2b**).

### The Resistance of Chimeras Treated with Glycyrrhizin to P. aeruginosa i.d. Infection

**Figure 3:**
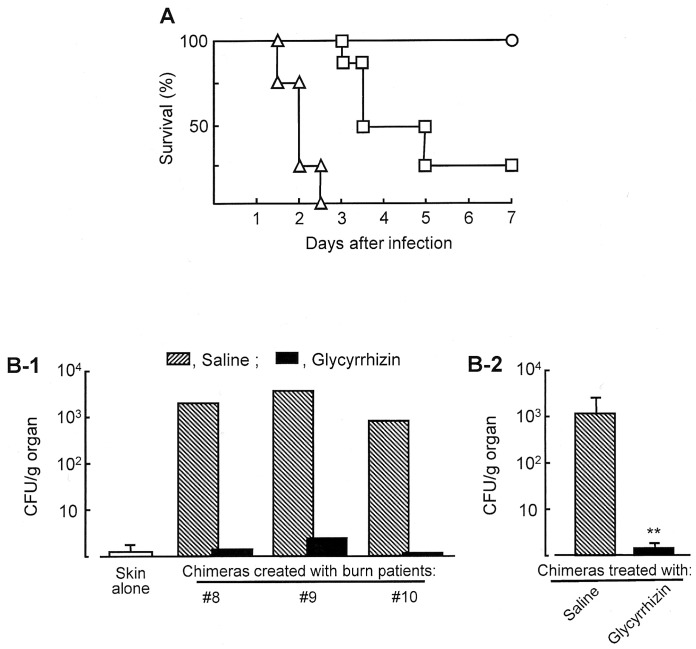
Effect of glycyrrhizin on the resistance of chimeras against sepsis stemming from *P. aeruginosa* i.d. infections. **A.** Survival of patient chimera substitutes i.d. infected with 20 LD_50_ of *P. aeruginosa*. The chimeras exposed to the pathogen were treated i.p. with 10 mg/kg (open circles) and 3 mg/kg (open squires) of glycyrrhizin or saline (0.2 ml/mouse, control, open triangles). Each infection experiment was performed by a group of 2–3 mice, and it was repeated 3 times. The results obtained were combined and displayed in the figure. **B.** Growth of pathogen in kidneys of patient chimeras i.d. infected with 20 LD_50_ of *P. aeruginosa*. Patient chimeras created with unburned skin tissues from patients #8∼#10 and their CD31^+^ IMC were exposed to the pathogen, and treated with glycyrrhizin (10 mg/kg) or saline (0.2 ml/mouse, control). Two days after infection, the growth of *P. aeruginosa* in kidneys of these chimeras was measured by a standard colony counting method. A white bar shows the results obtained from a group of mice grafted with skin alone. Fig. 3B-1 shows 3 independent experiments performed using skin and blood specimens from 3 patients, and Fig. 3B-2 shows mean ± SEM of the results shown in Fig. 3B-1. **P<0.01 vs saline-treated control.

After treatment with or without glycyrrhizin (10 mg/kg, i.p., twice), survival of patient chimera substitutes i.d. infected with 20 LD_50_ of *P. aeruginosa* was tested. As shown in **Fig. 3a**, all of the chimeras treated with saline died within 3 days of the infection, while 100% of the same chimeras treated with glycyrrhizin survived more than 7 days after the infection. The anti-pseudomonal effect of glycyrrhizin was further examined in patient chimeras. Patient chimeras created with unburned skin tissues from patients #7∼#10 and their CD31^+^ IMC were infected i.d. with 20 LD_50_ of *P. aeruginosa* at the grafting site 2 days after the CD31^+^ IMC inoculation. Then, they were treated i.p. with 10 mg/kg of glycyrrhizin or saline 6 and 24 hours after the IMC inoculation. Two days after the infection, kidneys were removed from the chimeras and homogenized in 2 ml PBS. The numbers of bacteria in the homogenates were measured by a standard colony counting method. As shown in **Fig. 3b**, bacteria grew in kidneys of all patient chimeras infected with *P. aeruginosa*. However, the pathogen was not detected significantly in kidneys of the chimeras treated with glycyrrhizin.

### The Suppressor Cell Activity of CD31^+^ IMC Treated with Glycyrrhizin

**Figure 4:**
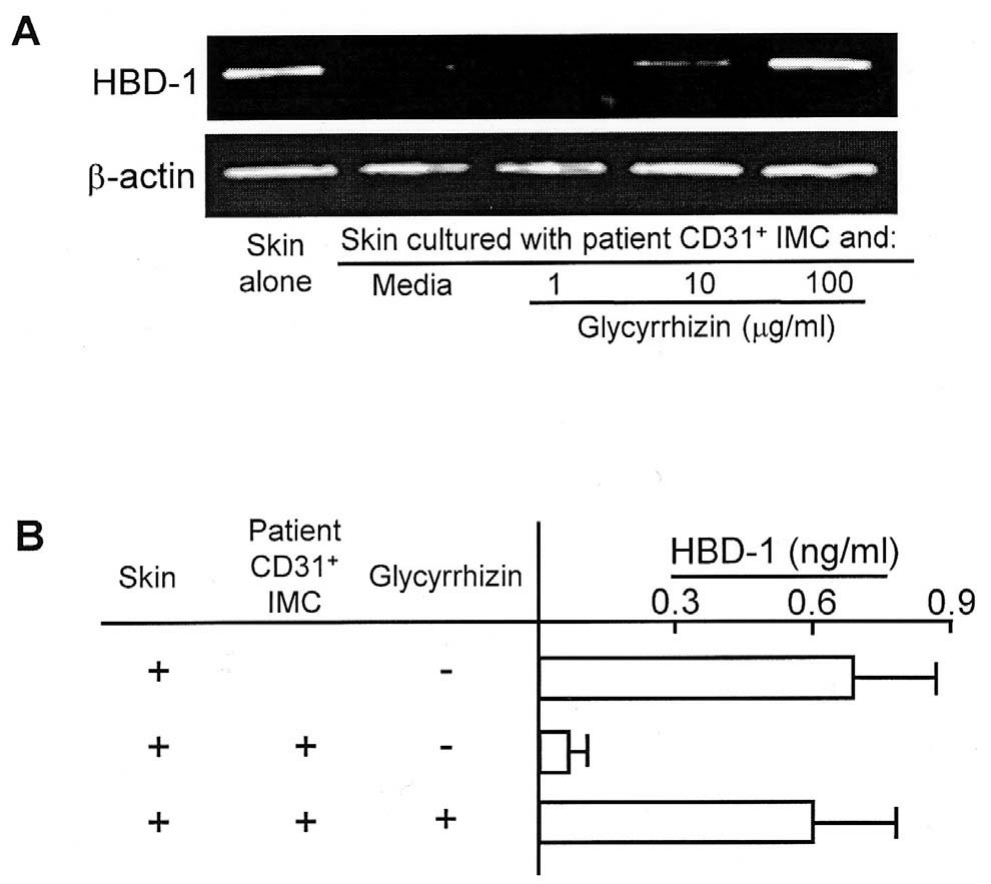
Effect of glycyrrhizin on the expression of HBD-1 mRNA and HBD-1 production by patient unburned skin tissues transwell-cultured with the same patient CD31^+^ IMC. **A.** mRNA expression. Patient #11 unburned skin tissues (0.2×0.2 cm, lower chamber) were transwell-cultured with their CD31^+^ IMC (1×10^5^ cells/ml, upper chamber) for 48 hours in the presence or absence of 1 to 100 µg/ml of glycyrrhizin. Skin tissues harvested were analyzed for HBD-1 mRNA by RT-PCR. Data are representative of 4 individual experiments using unburned skin tissues and CD31^+^ IMC obtained from patients #12∼#15. **B.** HBD-1 production. Transwell-cultures between unburned skin tissues from patients #11∼#15 and their CD31^+^ IMC were respectively performed in the presence of 100 µg/ml of glycyrrhizin. Culture fluids harvested 48 hours after cultivation were assayed for HBD-1 by ELISA.

The effect of glycyrrhizin on the suppressor cell activity of patient CD31^+^ IMC on HBD-1 production by patient unburned skin tissues was examined *in vitro*. CD31^+^ IMC (1×10^5^ cells/ml, upper chamber) from burn patients #11∼#14 were transwell-cultured with unburned skin tissues (0.2×0.2 cm, lower chamber) in culture media supplemented with or without 1 to 100 µg/ml of glycyrrhizin. Forty-eight hours after cultivation, skin tissues in the lower chamber were analyzed for HBD-1 mRNA by RT-PCR. Results obtained are shown in **Fig. 4a**. HBD-1 mRNA was not expressed by unburned skin tissues from patient #11 when the skin was transwell-cultured with patient CD31^+^ IMC. However, mRNA expression by the skin tissues was displayed when the same transwell-cultures were performed with 10 or 100 µg/ml of glycyrrhizin. Similar results were obtained when experiments were repeated utilizing unburned patient skin tissues from patients #12∼#14.

In addition, amounts of HBD-1 in the culture fluids obtained from the above transwell-cultures were measured by ELISA. HBD-1 production by unburned skin tissues from burn patients #11∼#14 was inhibited when it was transwell-cultured with patient CD31^+^ IMC. However, HBD-1 was produced when the same transwell-cultures were performed with 100 µg/ml of glycyrrhizin (**Fig. 4b**). These results shown in Figs. 4A (mRNA expression) and 4B (protein production) indicate that HBD-1 is produced by patient unburned skin tissues in transwell-cultures performed with patient CD31^+^ IMC and glycyrrhizin.

**Figure 5:**
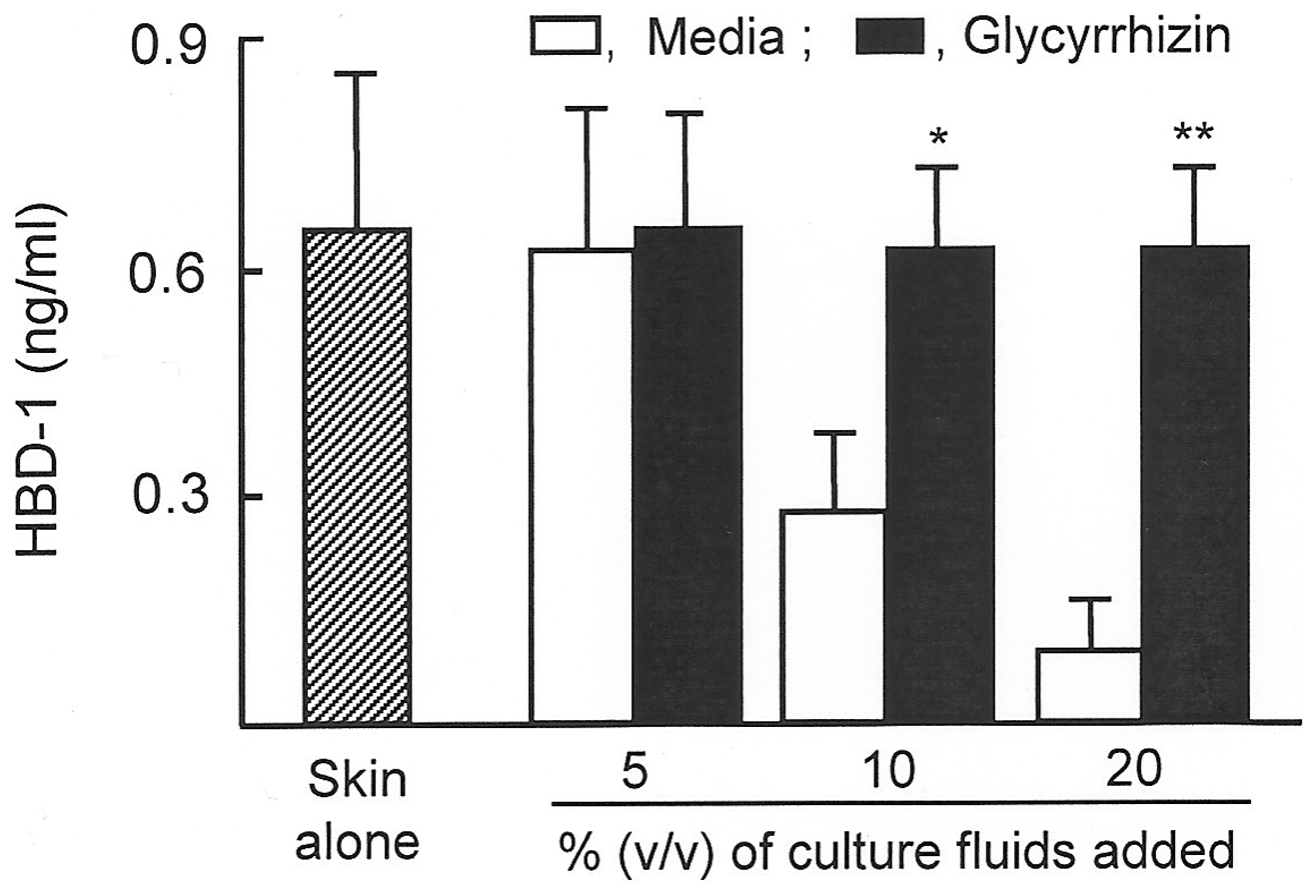
HBD-1 production by patient unburned skin tissues in cultures supplemented with CD31^+^ IMC culture fluids. Patient #16∼#18 CD31^+^ IMC (1×10^6^ cells/ml) were individually treated with or without glycyrrhizin (100 µg/ml) for 12 hours. After washing with media, these cells were cultured for an additional 36 hours. Culture fluids harvested from the cultures supplemented with or without glycyrrhizin were added (5∼20%, v/v) to cultures of patient unburned skin tissues (0.2×0.2 cm), and cultured for 48 hours. Culture fluids harvested were assayed for HBD-1 by ELISA. *P<0.05; **P<0.01 vs. cultures without glycyrrhizin.

In our accompanying paper [10], culture fluids of patient CD31^+^ IMC (10–20%, v/v) have been shown to be inhibitory on HBD-1 production by NHEK. Therefore, the culture fluids harvested from cultures of patient #16 CD31^+^ IMC that were previously treated with glycyrrhizin were tested for their inhibitory activities on HBD-1 production by patient unburned skin tissues. HBD-1 production was inhibited by 55% to 80%, when 10% to 20% (v/v) of culture fluids were added to the cultures of patient unburned skin tissues. When culture fluids were obtained from patient CD31^+^ IMC that were previously treated with glycyrrhizin, the suppressor activities of the culture fluids were not demonstrated. Similar results were obtained when experiments were performed with CD31^+^ IMC from patients #17 and #18. Results shown in **Fig. 5** display the average HBD-1 production.

**Figure 6:**
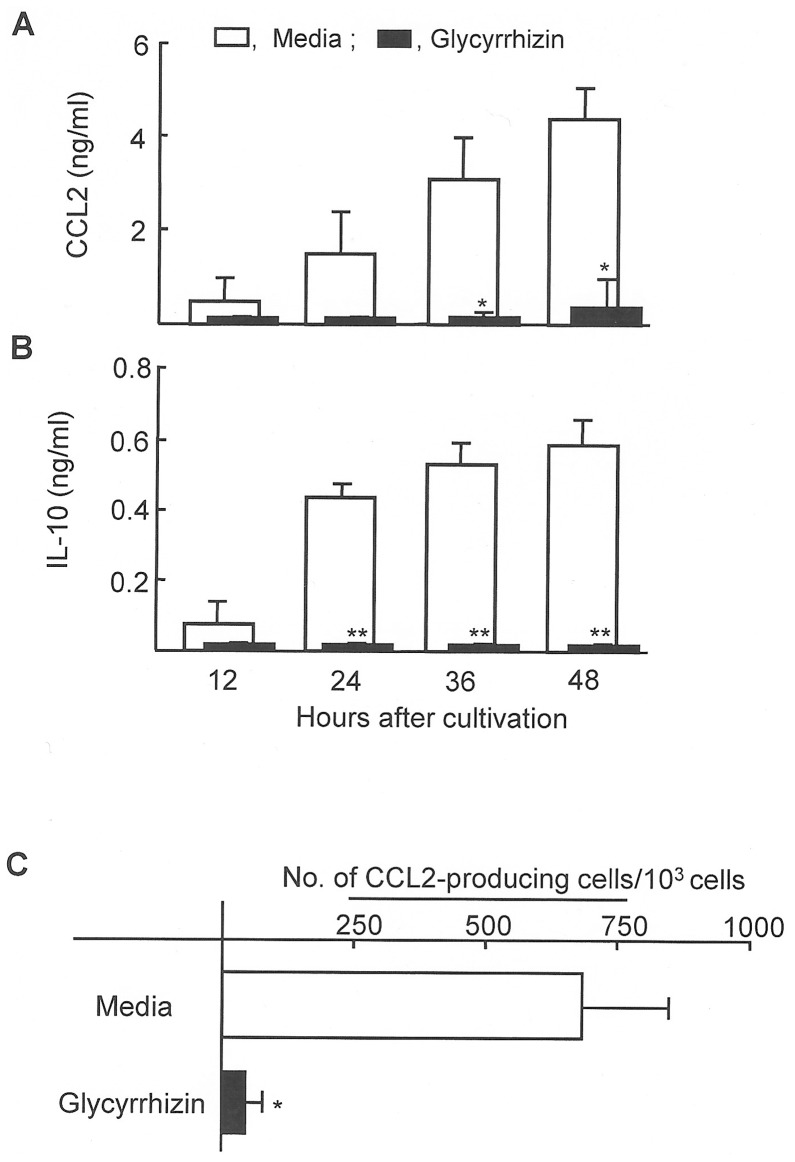
Effect of glycyrrhizin on CCL2 and IL-10 production by patient CD31^+^ IMC. CCL2 (**A**) and IL-10 (**B**) production by patient CD31^+^ IMC in cultures supplemented with glycyrrhizin. Patient #16∼#18 CD31^+^ IMC (1×10^5^ cells/ml) were cultured in the presence or absence of glycyrrhizin (100 µg/ml) for 12 hours. Then, these cells were washed with media, and cultured for an additional 12 to 48 hours. Culture fluids harvested were assayed for CCL2 and IL-10 by ELISA. *P<0.05; **P<0.01 vs control. **C.** Effect of glycyrrhizin in the numbers of CCL2-producing cells in cultures of patient CD31^+^ IMC. In cultures, patients #16∼#18 CD31^+^ IMC were treated with glycyrrhizin (100 µg/ml) for 48 hours. Then, numbers of CCL2-producing cells in the cultures were determined by ELISPOT assay. *P<0.05 vs control.

In our accompanying paper [10], CCL2 and IL-10 released from patient CD31^+^ IMC were shown to be effector molecules for the inhibition of HBD-1 production by NHEK. Therefore, in the following studies, the effect of glycyrrhizin on the production of CCL2 and IL-10 by patient CD31^+^ IMC was examined. Thus, patient #16 CD31^+^ IMC were cultured with or without 100 µg/ml of glycyrrhizin for 12 hours. Then, these cells were washed with media and cultured for an additional 12 to 48 hours. Culture fluids harvested were assayed for CCL2 and IL-10 by ELISA. As a result, significant amounts of CCL2 and IL-10 were not detected in culture fluids of patient #16 CD31^+^ IMC treated with glycyrrhizin, while these soluble factors were detected in cultures of patient CD31^+^ IMC not treated with glycyrrhizin. Similar results were obtained when the experiments were performed with CD31^+^ IMC from patients #17 and #18 (**Figs. 6a and 6b**). In addition, the number of CCL2-producing cells in the patient CD31^+^ IMC preparation treated with or without glycyrrhizin was enumerated using ELISPOT assay. On average, 692 CCL2^+^ cells per 1000 CD31^+^ IMC were counted in CD31^+^ IMC preparations from patients #16∼#18, whereas only on average, 50 CCL2^+^ cells were detected in the same IMC preparations treated with glycyrrhizin (**Fig. 6c**).

## Discussion

Severely burned patients with decreased host antibacterial defenses are highly susceptible to various infections [1]–[3]. Antibiotics are effective against topical wound infections and subsequent development of sepsis in severely burned patients [3]. However, antibiotic chemotherapies often encourage abnormal microflora and multi-antibiotic-resistant bacterial generation. Therefore, a new paradigm to treat opportunistic burn wound infections is urgent for severely burned patients. In our murine studies, *P. aeruginosa* burn wound infection and subsequent sepsis have been immunologically controlled by glycyrrhizin [12]. This suggests that a glycyrrhizin therapy may be the new paradigm to treat severely burned patients with *P. aeruginosa* burn wound infection. To establish anti-pseudomonal efficacies of glycyrrhizin in a more clinical setting, in this paper, the protective effect of glycyrrhizin on sepsis stemming from *P. aeruginosa* skin infections was studied in a chimera model of severely burned patients. The experiments were performed in γ-irradiated NOD-SCID IL-2rγ^null^ mice grafted with unburned skin tissues (discarded small pieces of skin that were utilized for autograft surgery) of severely burned patients and their syngeneic peripheral blood CD31^+^ IMC (patient chimeras). Also, in one part of the infection experiments, patient chimera substitutes (γ-irradiated NOD-SCID IL-2rγ^null^ mice grafted with cultured NHEK and experimentally induced CD31^+^ IMC) were utilized, because sufficient numbers of burn patient peripheral blood CD31^+^ IMC were not available. Immunological similarities of patient chimeras and patient chimera substitutes have been demonstrated by (i) impaired production of human skin antimicrobial peptides, (ii) increased susceptibility to *P. aeruginosa i.d.* infection, and (iii) remarkable production of human CCL2 and IL-10 (effector factors for patient CD31^+^ IMC to inhibit antimicrobial peptide production).

Based on our previous studies [12], [22], glycyrrhizin was administered i.p. to these chimeras at a dose of 1 to 10 mg/kg. The greatest efficacy of glycyrrhizin on recovering HBD-1 production was seen in the chimeras treated with a 10 mg/kg dose of glycyrrhizin. The protective effect of glycyrrhizin against graft site *P. aeruginosa* i.d. infection was examined in patient chimeras that were previously eliminated of murine skin antimicrobial peptides. This syngeneic experimental system may display accurate antibacterial responses shown by severely burned patients with *P. aeruginosa* burn wound infection. After infection with *P. aeruginosa* at the grafted site, bacteria grew in kidneys of the chimeras, while bacteria were not detected in kidneys of the same chimeras treated with glycyrrhizin. These results indicate that sepsis stemming from grafted site *P. aeruginosa* infection is not developed in patient chimeras treated with glycyrrhizin. The effect of glycyrrhizin on the survival of patient chimera substitutes i.d. infected with a lethal dose of *P. aeruginosa* was also tested. As a result, all of the chimeras treated with saline died within 3 days of infection, but 100% of the same chimeras treated with glycyrrhizin survived more than 7 days after the same infection. The results obtained in the chimera studies strongly indicate that glycyrrhizin is beneficial to treat pseudomonal burn wound infection and subsequent sepsis in severely burned patients.

Currently, intravenous doses of glycyrrhizin (80–120 mg/day) is clinically utilized in patients with chronic hepatitis [35]. Most individuals treated with high doses of glycyrrhizin (400 mg or more) experience some adverse effects, such as hypermineralocorticoidism with sodium retention and potassium loss, edema, increased blood pressure and depression of the rennin-angiotensin-aldosterone system [36]. These adverse effects are due to the hydrolysis of glycyrrhizin in the intestine to the pharmacologically active compound glycyrrhetic acid, which inhibits the enzyme 11 beta-hydroxysteroid dehydrogenase that is involved in the metabolism of corticosteroids. Inhibition of this enzyme leads to increased cortisol levels in the kidneys [36]. In our studies, however, non-toxic doses of the compound are shown to be active in the protection of chimeras infected with a lethal dose of *P. aeruginosa* in grafted site tissues. For the clinical utilization of glycyrrhizin against burn wound infections in severely burned patients, more detailed toxicology tests are required.

Glycyrrhizin did not have any direct action on the growth of *P. aeruginosa.* Therefore, the protective effect of glycyrrhizin against grafted site pseudomonal infections in chimeras must be displayed through the host’s antibacterial functions influenced by glycyrrhizin. In previous murine studies [12], glycyrrhizin-associated improvement on antimicrobial peptide production is shown to be key on the protection of severely burned mice with pseudomonal wound infections. The importance of skin antimicrobial peptides against invasion of pathogens in wound tissues has been well-described [4]–[7]. However, antimicrobial peptides are not produced in skin tissues surrounding the burn wound [8], [11], and small numbers of the pathogen that invaded the burn wound can spread easily into the whole body [11]. Skin antimicrobial peptides were produced in tissues surrounding the burn area of mice treated with glycyrrhizin [12]. Since glycyrrhizin had no stimulating activities on the antimicrobial peptide production by skin keratinocytes, glycyrrhizin may improve the peptide production by keratinocytes indirectly. Gr-1^+^CD11b^+^ IMC have been identified as murine effector cells on burn-associated impairment of the peptide production [11]. In this human study, CD31^+^ IMC have been identified as cells that inhibit the peptide production by skin keratinocytes. Human antimicrobial peptides were not produced by unburned skin tissues in transwell-cultures with their syngeneic CD31^+^ cells. In this transwell-culture, the effect of glycyrrhizin on the improvement of the peptide production was tested. In the results, the peptides were produced in this transwell-culture supplemented with glycyrrhizin. Subsequently, CCL2 and IL-10 released from CD31^+^ IMC were shown to be inhibitory on the peptide production by skin keratinocytes, and these cytokines were not produce by CD31^+^ IMC treated with glycyrrhizin. All of these facts strongly suggest that the inhibitory effect of glycyrrhizin on the production of IL-10 and CCL2 by CD31^+^ IMC may be important in the anti-pseudomonal activity of the compound.

How glycyrrhizin suppresses cytokine production by CD31^+^ IMC is not known. Recent studies have indicated that glycyrrhizin binds to the glucocorticoid-like receptor [37] and mobility group box protein 1 (HMGB1) [38]. Because the enhancer region of the CCL2 gene is inhibited by glucocorticoids, progesterone, and estrogen in cells stimulated with inflammatory mediators [39], [40], the binding of glycyrrhizin to the glucocorticoid-like receptor may result in the inhibition of CCL2 gene activation. The phosphorylation of HMGB1 by casein kinase I and protein kinase C promotes the release of inflammatory cytokines; however, the phosphorylation of HMGB1 is inhibited by glycyrrhizin after binding [38]. These inhibitory effects of glycyrrhizin on the transcriptional factors may be involved in controlling cytokine production by CD31^+^ IMC. Furthermore, the inhibitory effects of glycyrrhizin on the activation of NF-κB, STAT3 and MAPKs (including JUNK, p38 and ERK) in monocytes/macrophages have been demonstrated during acute inflammatory responses [41]–[46]. The activation of NF-κB and MAPKs is important for CCL2 production [47], and the increased production of CCL2 requires STAT3 activation [48]. These molecular mechanisms influenced by glycyrrhizin may also be involved in suppressing cytokine production by CD31^+^ IMC. Further analytical studies are required.

CD31^+^ IMC may function to suppress the burn-associated inflammation through the production of IL-10. This suggests that the time course of burn wound healing may be influenced by these cells. In our experiments, CD31^+^ IMC are shown to suppress host antibacterial defense through the production of CCL2 and IL-10, and glycyrrhizin has improved host antibacterial resistance through the inhibition of CCL2 and IL-10 production by CD31^+^ IMC. To determine how wound healing is influenced by the glycyrrhizin treatment, further studies are required.

## Supporting Information

Figure S1 Resistance of γ-irradiated NOD-SCID IL-2rγ^−/−^ mice treated with anti-AMP IgG to *P. aeruginosa* skin infection. γ-Irradiated NOD-SCID IL-2rγ^−/−^ mice were treated with s.c. with (solid circles, 10 mice) or without anti-murine AMP IgG (open circles, 10 mice) and infected i.d with 10 (A), 10^2^ (B), 10^3^ (C) and 10^4^ (D) of *P. aeruginosa*.
(TIF)
