# Supplementary figures and images for: Effect of Glycyrrhizin on Pseudomonal Skin Infections in Human-Mouse Chimeras
Source: PLoS One. 2014 Jan 30;9(1):e83747. doi: 10.1371/journal.pone.0083747 (PMC3907411; doi:10.1371/journal.pone.0083747)

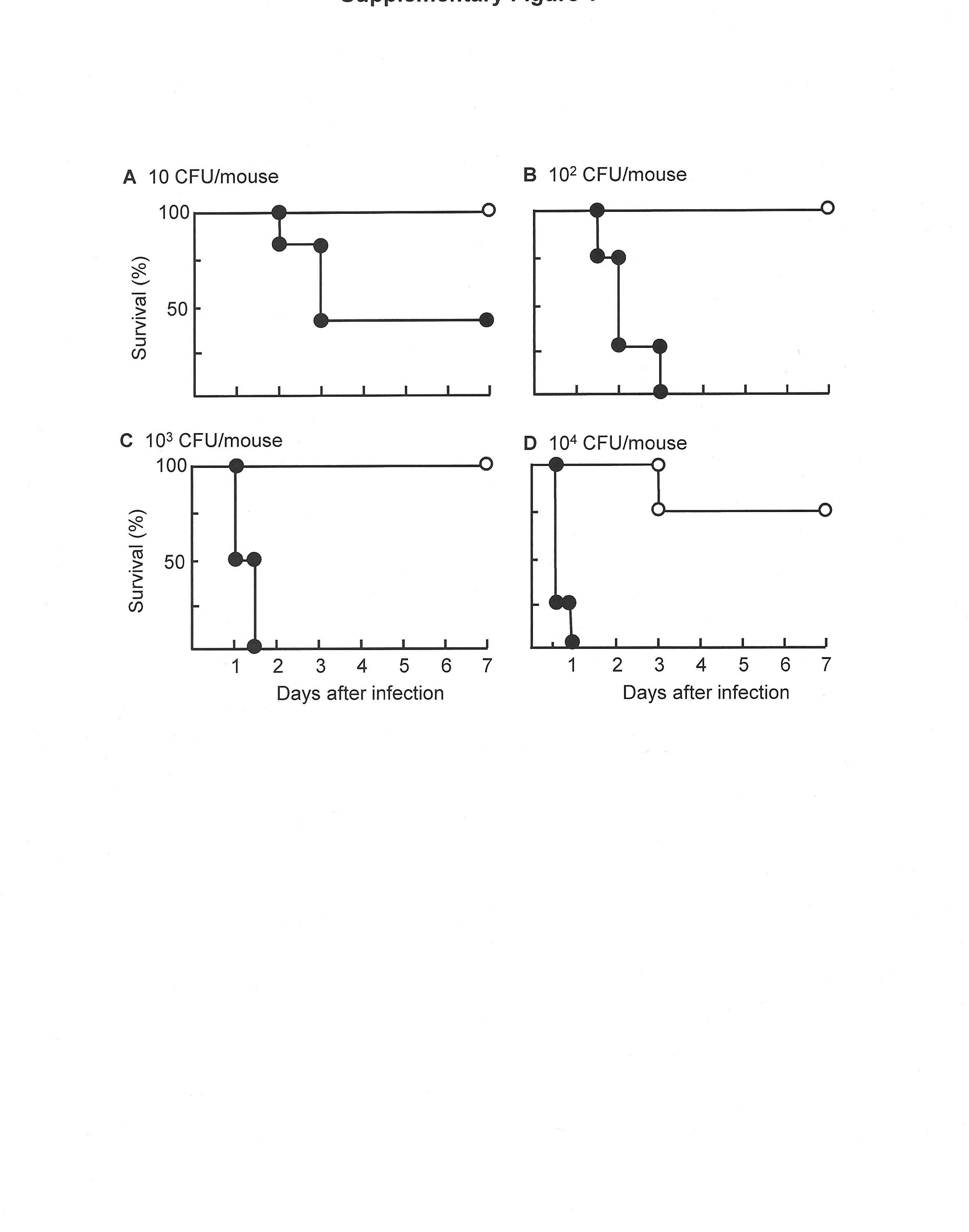

Supplement: Figure S1 — Resistance of γ-irradiated NOD-SCID IL-2rγ−/− mice treated with anti-AMP IgG to P. aeruginosa skin infection. γ-Irradiated NOD-SCID IL-2rγ−/− mice were treated with s.c. with (solid circles, 10 mice) or without anti-murine AMP IgG (open circles, 10 mice) and infected i.d with 10 (A), 102 (B), 103 (C) and 104 (D) of P. aeruginosa. (TIF) [file pone.0083747.s001.tif]
